# Animal movement and associated infectious disease risk in a metapopulation

**DOI:** 10.1098/rsos.220390

**Published:** 2023-02-01

**Authors:** Daniella J. Dekelaita, Clinton W. Epps, David W. German, Jenny G. Powers, Ben J. Gonzales, Regina K. Abella-Vu, Neal W. Darby, Debra L. Hughson, Kelley M. Stewart

**Affiliations:** ^1^ Department of Fisheries and Wildlife, Oregon State University, Corvallis, OR 97331, USA; ^2^ Sierra Nevada Bighorn Sheep Recovery Program, California Department of Fish and Wildlife, Bishop, CA 93514, USA; ^3^ Biological Resources Division, National Park Service, 1201 Oakridge Drive, Fort Collins, CO 80525, USA; ^4^ Wildlife Investigations Laboratory, California Department of Fish and Wildlife, 1701 Nimbus Road, Rancho Cordova, CA 95670-4503, USA; ^5^ Wildlife Branch, California Department of Fish and Wildlife, 1812 Ninth Street, Sacramento, CA 95811, USA; ^6^ Mojave National Preserve, National Park Service, 2701 Barstow Road, Barstow, CA 92311, USA; ^7^ Department of Natural Resources and Environmental Science, University of Nevada, Reno, NV 89557-0186, USA

**Keywords:** animal movement, bighorn sheep, connectivity, infectious disease, metapopulation, transmission risk

## Abstract

Animal movements among habitat patches or populations are important for maintaining long-term genetic and demographic viability, but connectivity may also facilitate disease spread and persistence. Understanding factors that influence animal movements is critical to understanding potential transmission risk and persistence of communicable disease in spatially structured systems. We evaluated effects of sex, age and *Mycoplasma ovipneumoniae* infection status at capture on intermountain movements and seasonal movement rates observed in desert bighorn sheep (*Ovis canadensis nelsoni*) using global positioning system collar data from 135 individuals (27 males, 108 females) in 14 populations between 2013 and 2018, following a pneumonia outbreak linked to the pathogen *M. ovipneumoniae* in the Mojave Desert, California, USA. Based on logistic regression analysis, intermountain movements were influenced by sex, age and most notably, infection status at capture: males, older animals and uninfected individuals were most likely to make such movements. Based on multiple linear regression analysis, females that tested positive for *M. ovipneumoniae* at capture also had lower mean daily movement rates that were further influenced by season. Our study provides empirical evidence of a pathogenic infection decreasing an individual's future mobility, presumably limiting that pathogen's ability to spread, and ultimately influencing transmission risk within a spatially structured system.

## 1. Introduction

Movement of animals among populations is important to conservation of wildlife species because connectivity buffers against extinction from demographic stochasticity while maintaining genetic diversity and consequently promotes long-term population persistence and viability [1–3]. Migratory or nomadic behaviour in animals, however, can contribute to disease transmission [4,5], and thus connectivity may also maintain host–pathogen dynamics within a spatially structured system, and cause outbreaks or facilitate disease persistence [6,7]. As such, patterns of connectivity can potentially undermine efforts to conserve wildlife populations threatened by disease, although some studies alternatively suggest that migration and nomadism can reduce infection risk. For example, disease transmission may be prevented if uninfected individuals move away from high-risk areas, or if infected hosts recover or die during long-distance movements that can be energetically costly or otherwise hazardous [4,5,8,9]. We further consider the model of ‘parasite-induced migration stalling’, which is a positive feedback that results between increasing parasite burdens and reduced movement that has been modelled for migratory populations [4,10,11] and has been observed in some migratory birds [12]. Although our population was not migratory *per se*, individuals in our study made long-distance movements between mountain ranges. Therefore, we considered that the propensity to undertake long-distance movements may depend on the infection and recovery status of an animal as well. For example, individuals compromised by infection could be less inclined to travel long distances that would ultimately promote disease transmission and foster the spread throughout spatial networks.

Several studies have examined effects of parasite infection on host movement and have reported reduced locomotion and dispersal activity resulting from negative effects to host anatomy and resources [13–16]. Specifically, parasite-induced modifications to host morphology and physiology can have direct mechanical impacts on movement, or cause lower endurance and increased lethargy that inhibits movement [15]. Additionally, parasitism may impose a respiratory burden on the host that can also restrict movement and dispersal behaviour [17,18]. Studies have observed impacts of parasitism on migration movements as well, whereby parasitized animals demonstrated reduced performance capacity by engaging different migratory patterns than non-infected individuals, and not reaching or arriving late to spawning and breeding grounds [14]. We submit by extension that if an animal's movement activity is limited by infection, the transmission potential and survival of the disease agent within a spatial network may also be limited. Alternatively, depending on the relative timescales of movement and infection, if movement of infected individuals among populations occurs only slightly more frequently than the typical infectious period within a population, persistence of the disease could be increased [19]. Regardless, changes in movement rates of infected individuals are likely to affect disease transmission and persistence.

Metapopulations, which consist of spatially distinct groups, or populations, associated by proximity and connected through animal movements, can be defined by a network structure [20,21]. Metapopulations affected by disease therefore present a scenario whereby we can study animal movement trends and explore the potential transmission risk within a network system. We evaluated inter-population (i.e. intermountain) movements in a metapopulation of bighorn sheep (*Ovis canadensis*) following a pathogen-induced pneumonia outbreak in the Mojave Desert, California, USA in 2013 to identify factors influencing movements and gain a better understanding of potential transmission risk among populations. The Mojave Desert ecosystem features an assemblage of mountain ranges separated by low-lying areas that are fragmented by roads, freeways and other anthropogenic structures [22]. Populations of desert bighorn sheep (*O. c. nelsoni*) occupy many of these ranges (populations are typically identified by mountain range), and despite distance and fragmentation [23], a network maintained by occasional animal movements exists among these populations, allowing for gene flow and the persistence of metapopulation dynamics [24].

Pneumonia outbreaks in bighorn sheep populations typically result from direct contact between infected livestock (particularly domestic goats and sheep) and wild sheep, although once the disease is introduced, infected bighorn can spread pathogens within and among populations [25,26]. Populations seem to be most at risk of disease spread among individuals or new outbreaks derived from nearby conspecifics during periods or seasons when contact rates are high, for example during the breeding season when animals disperse, aggregate and commingle in high concentrations [25,27], and as such, transmission risk across populations may be influenced by seasonality as well. In May and June 2013, a pneumonia outbreak linked to the pathogen *Mycoplasma ovipneumoniae* occurred in the bighorn population at Old Dad Peak (Kelso Mountains) in the Mojave Desert, and infected animals were detected in neighbouring populations thereafter [28,29]. This pathogen is now thought to be the primary causal agent of bighorn respiratory pneumonia, despite the association of many other pathogens with that disease [30]. Prior to the outbreak, bighorn populations in the Mojave Desert of California were believed to be insulated from the threat of pneumonia because of reduced connectivity with neighbouring wild sheep systems and domestic herds, although prior exposure to *M. ovipneumoniae* has now been documented for some populations in the study [29]. *M. ovipneumoniae* is transmitted by direct contact among individuals, or through airborne transmission over short distances, but does not persist in the environment [26]. Infected individuals develop symptoms within days or weeks. For animals that survive, infections may clear within several months [31], although a subset of animals become chronically infected and can remain so for greater than 3 years [32]. To our knowledge, despite extensive research on respiratory disease in bighorn sheep, effects of microbial pneumonia on bighorn movement have not yet been studied.

Animal to animal pathogen transmission occurs when infected hosts contact and infect susceptible individuals [33]. Our objective was to assess contact potential and thereby potential pathogen transmission risk (i.e. the potential risk of transmission posed by an infected individual) between bighorn populations by evaluating intermountain movements (i.e. movements from one mountain range to another) with respect to sex, age, seasonality and *M. ovipneumoniae* infection status at the individual level. We assumed that individuals who were more likely to make intermountain movements were also more likely to be vectors of *M. ovipneumoniae* between populations, although infected animals could be less likely to make such movements because of decreased health. We had two hypotheses: *H1) Males are more likely to make intermountain movements, which are further dictated by age and seasonality*. Dispersal and long-distance movements have been observed more frequently in males than females [34–37], and given seasonal differences in behaviour and physiological requirements associated with reproductive phases within and between sexes [36,38–40], we predicted that time of year would further influence such movements and potential for pathogen spread. We also speculated that individuals greater than 5 years old would be more likely to make intermountain movements because males typically leave female groups when they are 2–4 years old [34,41] and increase travelling distances by an order of magnitude after 3–4 years old [42], and females behave more independently at 4–5 years old [43]. *H2) Bighorn sheep with active M. ovipneumoniae infections are less likely to make intermountain movements.* We assumed that animals who tested positive for *M. ovipneumoniae* at time of capture could experience adverse effects associated with infection that might inhibit long-distance movements, given that survival was lower for infected individuals, even years after capture [28]. Additionally, we included an analysis of daily movement rates for males and females, calculated from distances between daily locations, to further examine effects of seasonality, age and infection status on movement at a finer scale.

## 2. Material and methods

### 2.1. Study area

The Mojave Desert is a high-elevation desert, characterized by dry, hot summers and cold, wet winters, but also experiences late summer monsoons that account for at least 25% of the total annual rainfall [44,45]. Temperatures and precipitation vary with elevation; temperatures typically range from average lows of −1°C in the winter to average highs of 34°C in the summer and can exceed 40°C in the lower lying areas [46]. Mean annual precipitation is approximately 21 cm, with lower elevations receiving as little as 9 cm and higher elevations receiving as much as 25 cm annually [46]. We defined annual seasons based on a climograph for Mojave National Preserve [47], whereby October and November represented autumn, December through March represented winter, April through June represented spring and July through September represented summer.

**Figure 1.**
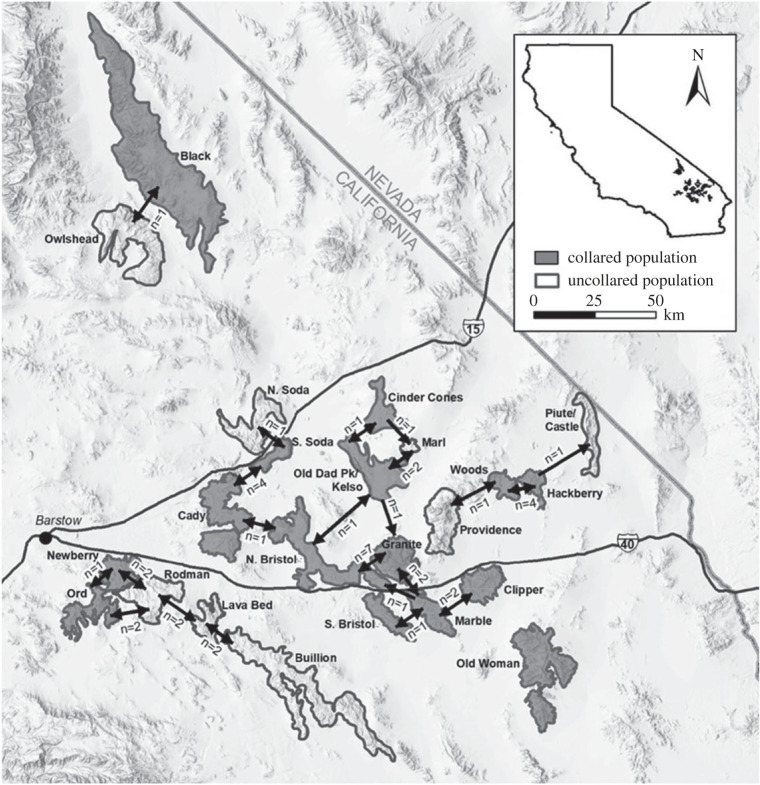
Intermountain movements (indicated by arrows) between bighorn sheep populations in the Mojave Desert, California, USA. Movements were detected using location data from animals with GPS radio-collars that were monitored from 2013 to 2018. Bidirectional arrows indicate two-way movements and unidirectional arrows indicate one-way movements; *n* denotes the number of animals that moved.

We studied individuals from 14 bighorn sheep populations between 2013 and 2018; populations were defined by mountain ranges and we assumed an individual belonged to the population within the range it was captured. We defined specific population boundaries following Epps *et al*. [22] and Creech *et al*. [48], where population boundaries were drawn on the basis of slopes greater than 10% given that bighorn sheep favour steep terrain, access to water, knowledge of past movement behaviour, and genetic differentiation. Distances among neighbouring populations vary from a few to dozens of kilometres (figure 1); genetic analyses indicate that intermountain movements decline with inter-population distance and rarely cross interstate highways [23,24]. Our study area encompassed ranges in the eastern and southern Mojave, which included Kelso, South Soda, Cady, North Bristol, South Bristol, Granite, Providence, Marble, Clipper, Hackberry, Woods, Piute, Newberry, Rodman, Ord, Old Woman and Bullion Mountains, located east of Barstow, California, south of Interstates 15 and 40, on lands managed by the Bureau of Land Management and National Park Service, Mojave National Preserve. Two additional ranges, Black and Owlshead Mountains, were located in the northern Mojave, north of Baker, California in Death Valley National Park.

The study area occurred between 36°00′00″ N and 34°15′00″ N and between 116°56′00″ W and 114°53′25″ W. Elevations ranged from approximately 300 m to 2000 m. Mountain ranges featured common desert scrub vegetation including catclaw acacia (*Senegalia greggii*), creosote bush (*Larrea tridentata*), rabbitbrush (*Ericameria paniculata*), brittlebush (*Encelia farinosa*), white bur-sage (*Ambrosia dumosa*), blackbrush (*Coleogyne ramosissima*), Mormon tea (*Ephedra* spp.), silver and buckhorn cholla (*Cylindropuntia echinocarpa* and *Cylindropuntia acanthocarpa*), Mojave yucca (*Yucca schidigera*), California barrel cactus (*Ferocactus cylindraceus*), and annual grasses and forbs that appeared seasonally in response to rainfall [49,50]. Common native mammal species included antelope ground squirrel (*Ammospermophilus leucurus),* black-tailed jackrabbit (*Lepus californicus*), desert cottontail (*Sylvilagus audubonii*), kit fox (*Vulpes macrotis*), coyote (*Canis latrans*), bobcat (*Lynx rufus*) and desert bighorn sheep. Mountain lion (*Puma concolor*), mule deer (*Odocoileus hemionus*) and free-ranging burro (*Equus asinus*) also occurred in some ranges, although burros are not native to the Mojave Desert.

### 2.2. Collaring, disease testing and ageing

Adult bighorn sheep were captured and fitted with global positioning system (GPS) radio-collars in November 2013, 2014, 2015 and March 2017 in the following ranges: Old Dad Peak/Kelso (*n* = 18), South Soda (*n* = 8), Cady (*n* = 10), North Bristol (*n* = 18), South Bristol (*n* = 19), Granite (*n* = 4), Marble (*n* = 26), Clipper (*n* = 13), Hackberry (*n* = 6), Woods (*n* = 6), Newberry (*n* = 2), Ord (*n* = 2), Old Woman Mountains (*n* = 1) and Black Mountains (*n* = 2). Animals were located aerially and captured using a net-gun fired from a helicopter [51], and were processed in the field following guidelines approved by the California Department of Fish and Wildlife and the National Park Service Institutional Animal Care and Use Committee (ACUP PWR_MOJA_Epps.Powers_DesertBighorn _2013.A3, 2013–2015), and as established by the American Society of Mammalogists for use of wild animals in research [52].

Nasal swabs were collected to determine infection status of individuals at time of capture and stored dry at −20 ^o^C prior to testing. Swabs were tested via polymerase chain reaction (PCR) to detect *M. ovipneumoniae* specific DNA sequences by Washington Animal Disease Diagnostic Laboratory (WADDL; Pullman, WA). Strain typing consisted of multi-locus sequence typing based on partial DNA sequences of the 16S-23S intergenic spacer region, the 16S ribosomal subunit, and RNA polymerase B and gyrase B genes, as described in Cassirer *et al*. [53]. Additionally, we aged animals and classified them as less than or greater than 5 years based on horn growth (i.e. number of horn annuli) and tooth eruption patterns [54–56].

### 2.3. Location data and movement metrics

Location data were obtained from animals fitted with store-on-board or GPS satellite collars: ATS G2110 (Advanced Telemetry Systems, Isanti, MN, USA), Lotek 4400 and Lifecycle (Lotek Wireless Inc., Newmarket, Ontario, Canada), Vectronic Survey (Vectronic Aerospace GmbH, Berlin, Germany) and Tellus Iridum 1D (Tellus GPS System-Followit AB, Lindesberg, Sweden). Collars were programmed to record locations between 1 and 10 times a day, varying by model type, and signalled mortality if they were motionless for more than 8 h. Data were either received through the Iridium satellite system (Iridium Communications, McLean, Virginia), Globalstar (Globalstar, Inc., Covington, Louisiana), or were downloaded when collars were recovered from animals.

We used adehabitatLT [57] in Program R [58] to produce movement metrics such as step-lengths, turning angles and time intervals [59–61] based on sequential location data for all animals. We identified and removed errant data points if the distance travelled was greater than mean ± 3 s.d. of the mean travel rate, the ratio of the distance travelled from three sequential points (i.e. 1, 2, and 3) for [1 to 2]/[2 to 3] was less than 0.9, and the turning angle between points was greater than 3 radians following Villepique *et al*. [62]. To reduce location error and minimize data reduction, we also discarded GPS locations if less than three satellites were used to obtain a fix, presumably retaining locations with mean error less than 40 m [63].

### 2.4. Movement modelling

#### 2.4.1. Intermountain movement analysis

We identified intermountain movements based on location data indicating that an animal had moved to a new mountain range, even if temporarily, and classified those individuals as movers. All statistical analyses were performed in Program R [58]. We modelled intermountain movement as an individual response (i.e. whether or not an animal had made at least one intermountain movement) using logistic regression with a binomial distribution and logit link; models were fit with the glm function. We assigned a value of 1 to movers (i.e. animals that made at least one intermountain movement, *n* = 27, see Results) and 0 to non-movers (i.e. animals that did not make any intermountain movements, *n* = 108, see Results), and evaluated models with covariates for PCR status at capture, sex and age. We tested correlations among covariates using the Pearson correlation coefficient. Because of concerns with sample size, we did not explore interactions. We modelled infection status as a continuous indicator variable whereby individuals received covariate values of 1 if they were PCR-positive for *M. ovipneumoniae* (i.e. positive for *M. ovipneumoniae* infection) at time of capture, values of 0 if they were negative, and a mean value of 0.5 if a test result was indeterminate based on results from WADDL, or if infection status was otherwise unknown due to missing data [28]. Sex and age were also modelled as indicator variables, whereby females received a covariate value of 0 and males received a covariate value of 1, while individuals less than 5 years old received a covariate value of 0 and individuals greater than or equal to 5 years old received a value of 1. Animals that became older than 5 years during the study were moved from the former cohort to the latter upon ageing out (capture dates were used to mark yearly intervals for ageing); we note that the method used for ageing yields minimum estimates [38]. Because lower survival rates in infected animals could bias our assessment of the effect of infection on intermountain movements during the study, we also tested for differences in dataset lengths (i.e. observation periods) for individuals in the three infection categories using a one-way ANOVA.

**Figure 2.**
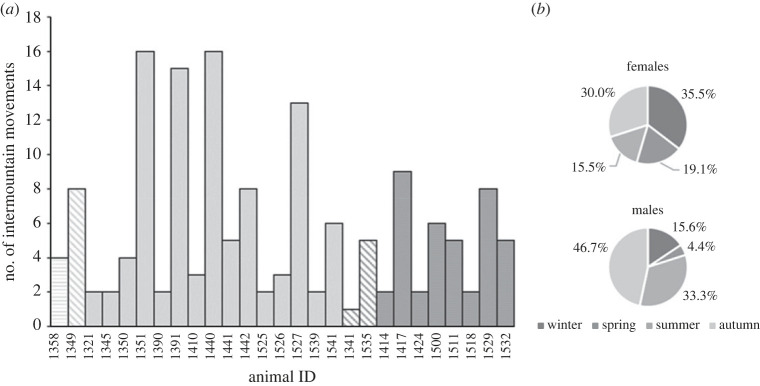
(*a*) Number of intermountain movements by individual for the 27 bighorn sheep that made such movements, out of 135 (27 males, 108 females) monitored (light grey = female movements, dark grey = male movements, hatches = individuals who tested positive for *Mycoplasma ovipneumoniae* at time of capture, horizontal lines = unknown infection status at the time of capture, solid = negative infection status at time of capture), and (*b*) proportions of bighorn intermountain movements occurring by season for males and females in the Mojave Desert, California, USA from 2013 to 2018 following a pneumonia outbreak.

To examine seasonal effects, we first calculated proportions of intermountain movements by annual season for males and females, which allowed us to identify seasonal differences reflecting biologically relevant seasons (figure 2*b*). For males, we identified biologically relevant seasons based on breeding from July to November and non-breeding from December to June, and for females based on mid-gestation to lambing (i.e. peak of lactation [36]) from October to April and post-lambing to early-gestation from May to September, as observed in desert bighorn sheep [36,64,65]. We tested the statistical significance of apparent seasonal differences in the number of movements associated with biological seasons for males (i.e. breeding versus non-breeding) and females (i.e. mid-gestation to lambing versus post-lambing to early-gestation) using Welch's *t*-test.

#### 2.4.2. Daily movement rate analysis

Additionally, we used standard linear regression to test effects of biological season, age, and PCR status at capture on daily movement rates in separate analyses for males and females, in order to further investigate movement behaviour regardless of intermountain activity. To generate daily movement rates, radio-collar data were resampled to one location per day for all individuals by removing extraneous locations such that consecutive locations for each animal were between 20 and 28 h apart. In cases where locations were missing and time lags exceeded 28 h, we retained the next location to allow for the next daily time step. We calculated daily movement rates from step-lengths (i.e. Euclidean distance between two consecutive locations measured in meters) divided by time lags between resampled locations, and multiplied rates by 24 h to yield metres per day (m/day; see electronic supplementary material, figure S1 for raw daily step-length distributions). Daily movement rates were then partitioned by biological season and averaged to produce seasonal mean daily movement rates for each individual. We modelled seasonal effects categorically, whereby each animal had two responses (i.e. one for each season) and received seasonal covariate input values of 1 for mean movement rates associated with breeding (for males) and mid-gestation to lambing (for females) and values of 0 for alternate periods. Age and PCR status were modelled as described in the intermountain movement analysis. We included a random intercept for each individual in our most strongly supported models to account for individual variation and potentially improve model fit [66]. We fit fixed-effects models with the lm function in Program R [58] and mixed-effects models with the lme4 package [67].

For both the intermountain movement and seasonal movement rate analyses, we ranked models using Akaike's Information Criterion adjusted for small sample sizes (AICc) [68,69] with the AICcmodavg package [70]. We considered the effects of all variables in our top models (*Δ*AICc scores less than 2) and interpreted covariate effects based on 90% confidence intervals (CIs) for a conservative evaluation of parameter importance [71,72]. For the intermountain movement analysis, we applied area under the receiver operating characteristic (ROC) curve (AUC) to evaluate goodness-of-fit of our top model [73] using LogisticDx [74]. For the seasonal movement rate analyses, we interpreted goodness-of-fit of our top models based on the coefficient of determination (*R*^2^), which we calculated using the MuMIn package [75]. We assessed normality and equal variance of residuals via quantile and residual plots using the olsrr package [76]. We also assessed normality of the random effect using a normal quantile plot for mixed-effects models, which we generated with the qqnorm function.

## 3. Results

We used data from 135 radio-collared bighorn (27 males and 108 females) from 14 populations in the Mojave Desert (figure 1) to evaluate movement trends with respect to sex, age, season and PCR status at capture between November 2013 and December 2018. We collected greater than 1 year of data from 128 individuals in our sample, and greater than 6 months to 1 year of data from the remaining 7 individuals (collars had an average lifespan of 2 years). Across our study, 27 individuals (10 males and 17 females) made 156 intermountain movements (males: 45, females: 111; figure 2*a*) and 108 individuals (17 males and 91 females) did not make intermountain movements. Based on proportions of intermountain movements occurring by season, there appeared to be a seasonal bias whereby most intermountain movements occurred during autumn (30%) and winter (35%) for females, which coincided with the period of mid-gestation to lambing, and during autumn (47%) and summer (33%) for males, which coincided with the breeding season. Lowest proportions of intermountain movements occurred in spring (19%) and summer (15%) for females, and in spring (4%) and winter (16%) for males (figure 2*b*). Differences across biologically relevant seasons for females and males were statistically significant based on Welch's *t*-test. Among movers, the mean number of intermountain movements per female during mid-gestation to lambing over the study period was 4.5 and during post-lambing to early-gestation was 2.0 (*t*_[24.2]_ = 2.1, *p* = 0.048). The mean number of intermountain movements per male (among movers) during breeding over the study period was 3.6 and during non-breeding was 0.9 (*t*_[11.9]_ = 2.5, *p* = 0.028). Thus, movement averages per animal-year were 1.3 for females and 0.9 for males.

Additionally, we did not detect any definitive dispersal movements (i.e. one-way movements away from source populations [77]), in that all intermountain movements were typically followed by return trips back to the mountain range of origin (figure 1), with some individuals making multiple round-trip movements over the study period. In three instances, animals died before returning to the mountain range of origin, but only after making multiple round-trip movements. In one other instance, an animal moved from the mountain range of origin to a neighbouring mountain range on the same day it was captured, and made no other intermountain movements during the study; we suspected that this animal may have originally been from the neighbouring range and likely returned immediately after being collared. All animals that made intermountain movements displayed long-range movement spurts whereby movements between mountain ranges were completed in 1–3 days and occurred at speeds as high as 16.4 km day^−1^. We classified one-way movements across multiple ranges during a less than two-week period as a single intermountain movement.

In the intermountain movement analysis, our top model indicated that age, sex and PCR status at capture were all important variables influencing intermountain movements (table 1). Age was positively correlated with intermountain movements (*β* = 1.06, 90% CI [0.24, 1.88]), whereby the odds of an individual greater than or equal to 5 years old making an intermountain movement were 2.89 times higher than an individual less than 5 years old. The effect associated with being male was also positive (*β* = 1.17, 90% CI [0.35, 1.99]), whereby the odds of a male making an intermountain movement were 3.22 times higher than a female. Lastly, the apparent effect of positive infection status was negatively correlated with intermountain movements (*β* = −1.33, 90% CI [−2.38, −0.28]) such that the odds of an individual undertaking an intermountain movement were 74% less if the individual was PCR-positive for *M. ovipneumoniae* at capture. The goodness-of-fit test for this model indicated acceptable predictability (AUC = 71.8%, 95% CI [62.9%, 80.7%]). Pearson correlation coefficients indicated no statistical support for relationships between PCR : age (*r* = −0.052, *p* = 0.5), PCR : sex (*r* = −0.15, *p* = 0.07) and age : sex (*r* = −0.034, *p* = 0.7). Furthermore, the length of the GPS collar datasets did not differ among individuals with different infection status categories at capture (ANOVA, *F*-ratio = 0.3011, *p* = 0.7405).

**Table 1.** Modelling results from an analysis evaluating intermountain movements of adult bighorn sheep in the Mojave Desert, California, USA from 2013 to 2018, following a pneumonia outbreak. Intermountain movements were detected using location data from animals that were captured and received GPS radio-collars in November 2013–2015 and March 2017. We tested effects of sex, age (i.e. less than or greater than 5 years old) and *Mycoplasma ovipneumoniae* infection (determined from PCR testing of nasal swabs collected from animals at time of capture) on intermountain movements as a binary response. Models were evaluated using Akaike's Information Criterion adjusted for small sample sizes (AICc).

model no.	model structure	*K*^a^	ΔAICc	*w_i_*^b^	LL^c^
1	age + PCR status + sex	4	0.00	0.58	−64.53
2	PCR status + sex	3	2.97	0.13	−67.07
3	age + PCR status	3	3.24	0.11	−67.21
4	age + sex	3	3.33	0.11	−67.25
5	PCR status	2	5.45	0.04	−69.35
6	sex	2	6.60	0.02	−69.92
7	age	2	8.11	0.01	−70.68
8	null	1	10.73	0.00	−73.02

^a^Number of model parameters.

^b^Akaike model weight.

^c^Log-likelihood.

Based on parameter estimates from our top model (and the intercept *β*_0_ = −2.17, 90% CI [−2.95, −1.39]), the probability of a male greater than or equal to 5 years old and PCR-negative at capture making an intermountain movement was 52% (±12% s.e.), whereas the probability for a male greater than or equal to 5 years old and PCR-positive at capture was 22% (±12% s.e.; electronic supplementary material, figure S2). Alternately, for a male less than 5 years old and PCR-negative at capture, the probability of an intermountain movement was 27% (±10% s.e.), and the probability for a male less than 5 years old and PCR-positive at capture was 9% (±6% s.e.). By contrast, the probability of an intermountain movement for a female greater than or equal to 5 years old and PCR-negative at capture was 25% (±6% s.e.), whereas the probability for a female greater than or equal to 5 years old and PCR-positive at capture was 8% (±4% s.e.). Alternately, for a female less than 5 years old and PCR-negative at capture, the probability of an intermountain movement was 10% (±4% s.e.), and the probability for a female less than 5 years old and PCR-positive at capture was 3% (±2% s.e.).

In the movement rate analysis, our highest ranking fixed-effects model for females indicated that season and PCR status were important variables influencing mean daily movement rates (marginal *R^2^* = 0.08; table 2). The random intercept in the corresponding mixed-effects model, which accounted for individual variation, improved model fit (conditional *R^2^* = 0.40); the fixed intercept for this model was *β*_0_ = 1027.07 (m/day), s.e. = 27.44. The season of mid-gestation to lambing was negatively associated with mean daily movement rates (mixed: *β* = −108.83 [m/day], s.e. = 26.81). Positive PCR status at capture was also negatively associated with mean daily movement rates (mixed: *β* = −118.67 [m/day], s.e. = 41.61). Quantile and residual plots for our top model indicated that the assumptions of normality and equal variance had been met for linear regression and the random effect was normally distributed. We note that the age parameter was included in the second highest ranking fixed-effects model, but the CIs overlapped 0 indicating that age was an uninformative parameter. For males, our highest ranking fixed-effects model indicated that season was the only important variable influencing mean daily movement rates (marginal *R^2^* = 0.45; table 2). The random intercept accounting for individual variation in the corresponding mixed-effects model slightly improved model fit (conditional *R*^2^ = 0.50); the fixed intercept for this model was *β*_0_ = 910.16 (m/day), s.e. = 49.74. The breeding season was positively associated with mean daily movement rates (mixed: *β* = 480.74 [m/day], s.e. = 66.90) and parameter estimates for age and PCR status in top models were not statistically supported (i.e. 90% CIs overlapped 0). Quantile and residual plots for the highest ranking model indicated that the assumptions of normality and equal variance had been met for linear regression, and the random effect was normally distributed. Based on mean parameter estimates, females moved 109 m day^−1^ less on average during the season of mid-gestation to lambing (October–April) than during post-lambing (May–September), and females that were PCR-positive moved 119 m day^−1^ less than PCR-negative females on average regardless of season, whereas males moved 481 m day^−1^ more during the breeding season (July–November) than the non-breeding season (December–June) on average, regardless of PCR status and age.

**Table 2.** Modelling results from an analysis evaluating seasonal mean daily movement rates of adult female and male bighorn sheep in the Mojave Desert, California, USA from 2013 to 2018, following a pneumonia outbreak. Mean daily movement rates were calculated using location data from animals that were fitted with GPS radio-collars in November 2013–2015 and March 2017. We tested effects of age (i.e. less than or greater than 5 years old), *Mycoplasma ovipneumoniae* infection (determined from PCR testing of nasal swabs collected from animals at time of capture) and season on seasonal mean daily movement rates using standard linear regression. Seasons were defined by the period of mid-gestation to lambing (October–April) and post-lambing to early-gestation (May–September) for females and the breeding period (July–November) and non-breeding period (December–June) for males. Models were evaluated using Akaike's Information Criterion adjusted for small sample sizes (AICc).

sex	model no.	model structure	*K*^a^	ΔAICc	*w*^b^	LL^c^
female						
	1	season + PCR status	4	0.00	0.61	−1729.38
	2	season + age + PCR status	5	1.02	0.37	−1728.85
	3	PCR status	3	8.67	0.01	−1734.75
	4	season	3	8.72	0.01	−1734.78
	5	age + PCR status	4	9.68	0.00	−1734.22
	6	season + age	4	10.10	0.00	−1734.43
	7	null	2	16.85	0.00	−1739.86
	8	age	3	18.20	0.00	−1739.52
male						
	1	season	3	0.00	0.31	−405.52
	2	season + age	4	0.44	0.25	−404.59
	3	season + PCR status	4	0.49	0.24	−404.61
	4	season + age + PCR status	5	0.77	0.21	−403.55
	5	null	2	32.96	0.00	−423.11
	6	age	3	34.17	0.00	−422.61
	7	PCR status	3	34.20	0.00	−422.62
	8	age + PCR status	4	35.38	0.00	−422.06

^a^Number of model parameters.

^b^Akaike model weight.

^c^Log-likelihood.

## 4. Discussion

Our study revealed that positive *Mycoplasma ovipneumoniae* infection status was associated with lower subsequent intermountain movement activity among individuals in a metapopulation of bighorn sheep. This finding has implications for disease persistence within the system, if transmission between populations is dependent on infected individuals moving from one population to another. While host response to disease in general varies depending on the illness, our study provides empirical evidence to suggest that in some cases pathogenic infection may compromise an individual's future mobility and in turn limit the ability for a pathogen to spread within and across populations. Compromised mobility from microbial pneumonia in bighorn may be further corroborated by necropsy results that reveal severe lung pathology in animals infected with *M. ovipneumoniae* and other respiratory pathogens [78,79], suggesting a decrease in lung capacity for oxygen exchange and therefore diminished exercise tolerance that may carry over after infections are cleared.

Additionally, males were more likely to undertake intermountain movements than females and older animals were more likely than younger animals. These results are consistent with our hypotheses and are largely indicated by other studies as well [34,36,37,43,80]. Some studies suggest, however, that males less than 5 years old are as likely to disperse and move long distances as older males [34,41], and that such movements occur rarely to never in females [81,82]. In our study, the proportion of females that made intermountain movements was smaller than the proportion of males (16% versus 37%), but among the bighorn that moved, females made more intermountain movements on average during the study. Moreover, intermountain movements among both males and females could not be classified as dispersal movements, because all but one individual made round-trip movements. Bleich *et al*. [83] reported a similar pattern for female bighorn in the Mojave Desert, and concluded these movements were either migratory or exploratory.

Seasonality also appeared to influence movement activity in both sexes. Males were more likely to make intermountain movements during the breeding season, while females were more likely to make intermountain movements during the period of mid-gestation to lambing. Our movement rate analysis provided additional support for the apparent seasonal effect on intermountain movements by males, whereby males had higher daily movement rates during the breeding season, a finding supported in the literature [34,36,37,41,80]. As such, we conclude that higher proportions of intermountain movements and higher mean daily movement rates during breeding were likely a function of rutting behaviour in males. We note that the effect of infection on movement rate was not supported for males (i.e. 90% CI overlapped 0), which might have resulted from reduced statistical power, given a substantially smaller sample size of males than females.

For females, results from the movement rate analysis were contrary to what we expected based on the seasonal bias of intermountain movements. Females had lower mean daily movement rates associated with the period of mid-gestation to lambing, when intermountain movements were highest. Positive infection status at capture, however, had an apparent negative effect on daily movement rates in females, as with intermountain movements, which was consistent with our hypothesis. We speculate that daily movement rates were likely higher during the post-lambing period (May–September) because of forage availability becoming more limited and scattered during the dry season, when animals were also constrained by access to limited water. Several studies have similarly concluded that larger home ranges used by desert bighorn sheep in the summer are the result of widely scattered resources [80]. Alternately, the period of mid-gestation to lambing (October–April) coincides with the growing season in the Mojave Desert [84]; females may have had lower daily movement rates during this period because availability of resources was greater and animals likely did not have to move as far to acquire them. By similar reasoning, intermountain movements may have been higher for females during this period, because resource constraints were lifted. As such, we conclude that movement patterns in females may have been dictated by forage and water availability, and we speculate that nutrition and parturition status may have also influenced movement behaviour.

In terms of potential transmission risk, we submit that movement rates and home range size likely influence potential for contact between individuals and groups. For example, if home ranges are larger when resources are less abundant, home ranges may be more likely to overlap resulting in increased potential for contact among individuals and across groups; bighorn sheep are gregarious and thus are likely to interact even if densities were lower in such a case. Moreover, bighorn sheep typically live in sexually segregated social groups year-round. These groups vary in size and stability depending on time of year [34,36,85], and aggregations of males and females occur during the rut in August through November in the Mojave Desert [36]. Thus, given lower movement rates among females during the period of mid-gestation to lambing (October–April), when female groups were presumably more stable, it is reasonable to conclude that contact potential and mixing between groups likely decreased. Likewise, during post-lambing, when movement rates were higher and groups were less stable, contact potential and mixing between groups likely increased. For this reason, we suspect that potential transmission risk across female groups within a given mountain range may be higher during the post-lambing period. Conversely, we expect females to pose the highest potential transmission risk across mountain ranges in the Mojave Desert during the period of mid-gestation to lambing, when the frequency of intermountain movements among females was highest. For males, movement rates and frequency of intermountain movements were highest during the breeding season (July–November), and as such, we would expect the potential transmission risk posed by an infected male both within and across mountain ranges to be highest during this period, which is also when aggregations of males and females occur.

We did not consider effects of barriers on intermountain movements in this study because movement paths between mountain ranges were loosely approximated from location data and barriers are difficult to assess [24], but we submit that the level of connectivity between ranges is likely another factor influencing transmission potential across ranges as well. We note that the analysis of intermountain movements largely assumes that individuals were solitary, especially with respect to the apparent age effect. Alternately, the movement rate analysis, which did not support age as a factor influencing movement activity in either sex, may indicate that within ranges movement rates reflected group activity. We recognize, however, that there was a high level of unexplained variance (greater than or equal to 50%) associated with top models in the movement rate analysis. We suspect that given the stochastic nature of resource availability in the desert [86], much of the unexplained variance may have resulted from fluctuations in environmental conditions across seasons and years, which could not be addressed with our dataset. Moreover, we suspect that the variance among individual females may have resulted from additional biological factors (e.g. pregnancy or lambing status and nutritional condition) we were unable to observe and account for throughout the study, which may have also overwhelmed effects of age. We suspect that males were more similar to each other because they were not subject to the demands of parturition that can variably alter nutritional condition and movement capability in females.

Population substructuring (i.e. group living within populations), spatial distribution and social dynamics of bighorn sheep are all factors that can influence contact rates between individuals and may therefore largely influence how respiratory disease is communicated and spread within a population sharing the same range [26,37,85]. Those factors have indirect implications for disease spread across populations as well, but host response to infection may be the most important factor influencing transmission [87]. Our study suggests that potential transmission risk of *M. ovipneumoniae* within and across bighorn populations varies depending on time of year and sex of an individual, but positive infection status may also inhibit animal movement and presumably potential transmission risk for at least some period of time following testing. We conclude that connectivity between populations, which is essential for maintaining long-term genetic and demographic viability of populations [3,22,35,85], also enables disease transmission within a system, but viability of an infectious disease depends on impacts to the host as well [87]. A disease that limits a host's mobility may inherently decrease the potential transmission risk posed by an infected individual and ultimately reduce pathogen survivability. Indeed, because we did not know the infection status of individuals at the time that movements occurred, given that some individuals may have cleared infections after capture and others may have gotten infected, the negative effect associated with *M. ovipneumoniae* infection on movement was probably underestimated in our study. We speculate that there are tradeoffs to connectivity within a metapopulation affected by infectious disease, and in some cases, the benefits could outweigh the risks, especially if effects of the disease itself reduce its ability to spread. Additional studies would be needed to assess potential tradeoffs of connectivity among populations threatened by particular diseases and inform management strategies aimed at conservation and restoration of these wildlife populations.

## Data Availability

The data are provided in the electronic supplementary material [88].
